# Not all pre-registrations are equal

**DOI:** 10.1038/s41386-022-01418-x

**Published:** 2022-08-18

**Authors:** Sophie Waldron, Christopher Allen

**Affiliations:** 1 School of Psychology, Cardiff University, Cardiff, CF24 4HQ UK; 2 Department of Biosciences, Durham University, Durham, DH1 3LE UK

**Keywords:** Psychology, Neuroscience

There is growing recognition that troubling numbers of experiments fail to replicate in fields relevant to Neuropsychopharmacology, from neuroimaging [1] to animal behaviour [2]. We believe a counteraction to this, led by pharmacology, is increased emphasis on the distinction between exploratory and confirmatory scientific practices [3]. Exploratory research, where multiple methodologies and analyses are trialled, is vital for discovery. In contrast, confirmatory research requires that this flexibility is minimised to address a well-specified research question. We aim to highlight problems that arise when this boundary is blurred, and how a new vista of publishing formats generally help by nailing down this distinction. However, some formats can allow problematic flexibility to re-enter under a confirmatory guise.

**Fig. 1:**
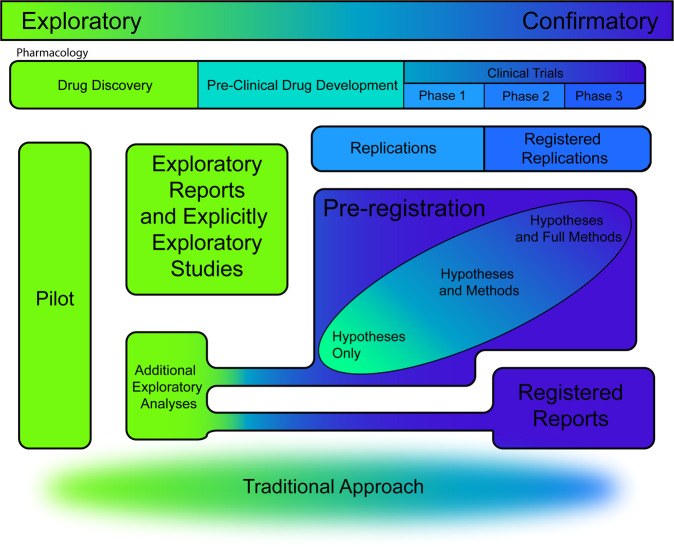
The range of publishing formats. Illustration of the diversity of publishing formats currently available in relation to the confirmatory/exploratory distinction colour coded as blue and green respectively, with gradients to show ambiguity between them.

Confirmation should follow exploration in science, as flexibility endemic in exploration can inflate effect sizes or generate false positives. Furthermore, confirmatory hypothesis testing is undermined when null-hypothesis based statistical methods are used to uncover unanticipated patterns in data, but results are tacitly presented as hypothesis-driven [4]. A mechanism to make exploratory and confirmatory research stages concrete and separate is pre-registration. Standard pre-registration entails a public record of experimental plans (e.g. Open Science Framework, OSF) before data collection or analysis. This ideally consists of well-specified aims, hypotheses, full methods including definition of variables, sampling, and analysis plans. Confirmatory pre-registration only works if flexibility in these components is minimised, but that may not always be the case (Fig. 1).

To assess flexibility in pre-registrations, we surveyed pre-registrations listed on the OSF after 2020 and pre-registered counterparts to published papers from 2019 and 2020 listed on Zotero (total *N* = 300, see https://osf.io/h8a6e/ for survey details and examples). This uncovered a range of completeness, spanning from notes on hypotheses only to fully specified, detailed experimental protocols with accompanying power analyses and sampling plans. Even when all elements are present, some pre-registrations were precise and exhaustive, while others gave brief details that could allow varying implementations, re-introducing problematic flexibility. For instance, brief descriptions of intended statistical tests and power calculations may be present but related to dependent variables that are not well specified. In addition to diversity in the form of pre-registration we also noted diversity in uptake across fields. As mentioned, pharmacology has a well-specified progression from exploration to confirmation [5]. In contrast, our survey found no examples of pre-registration in research using animals, where it has been specifically recommended as a way to reduce animal use by increasing publication of null findings and decreasing unnecessary replicates [6].

It could be argued that minimal pre-registration or hypothesis-only registration is better than nothing and may even be necessarily expedient for highly complex experiments. Anticipating all potential outcomes and practical contingencies is challenging. At least registration of hypotheses guards against changing hypotheses after seeing data or misremembering hypotheses. It also overcomes time-costs associated with full pre-registration, which can be particularly problematic for early career researchers on short contracts. Researchers might gain the perceived reliability benefits of pre-registration [7] without the drawbacks of longer and more involved research planning processes and the increased likelihood of null findings [8]. However, our concern is that minimal pre-registration reintroduces exploratory degrees of freedom and therefore an elevated risk of bias under a confirmatory banner. Worse, the public declaration of hypotheses could incentivise development of research methods toward demonstrating the hypotheses, rather than testing them. Therefore, minimal pre-registration means benefits might accrue for individuals but come at a cost for science.

Pre-registration templates, such as OSF Preregistration or AsPredicted, which require researchers to specify elements such as hypotheses and sampling procedures, appear to increase completeness [9]. Concerns about pre-registration flexibility are also greatly reduced through the publishing format Registered Reports [10]. Here flexibility is taken out of the hands of researchers and dictated by peer review prior to data collection or analysis. Final publication is then independent of data outcomes if registered methods are followed, further reducing publication bias toward positive results. A recent development here is the introduction of Peer Community In Registered Reports (PCI-RR, https://rr.peercommunityin.org/), which operates across a series of participating journals that commit to accepting editorial recommendations without further review. Here, following peer reviews, researchers and editors decide on the outlet.

Not all new formats make the exploratory/confirmatory distinction unambiguous. Science magazine has published articles under a ‘hypothesis’ banner that are linked to pre-registrations, but not formally via peer review. This offers opportunities for problematic flexibility under a hypothesis-driven label. Growing adoption of community consensus standards, such as those offered by PCI-RR, seem to offer a low-cost, open route to overcoming some of these ambiguities in publishing.

So long as details are well-specified, pre-registration can fit many study designs [11]. It is also worth emphasising that within pre-registered studies exploratory analyses based on observations during or after data collection are acceptable, so long as exploratory labelling is clear. Incremental pre-registrations that openly document experimental development from exploratory to confirmatory phases, or register multi-stage contingent experiments, are also acceptable and increasing in popularity.

Exploratory research is beginning to benefit from new publishing formats which aim to make the scientific discovery process open and well-documented. These make the level of exploration in research explicit [12], with continuous and open logging of hypothesis development and findings [13], and sequential procedures around the transition from exploratory to confirmatory phases [14]. These new formats are valuable as they draw outputs away from significant *p* values and positive research “stories” and toward narratives that fit the research.

‘Exploratory’ should not be seen as a negative label, and the label of ‘pre-registration’ does not necessarily mean elimination of flexibility. For pre-registration, until we reach a broad consensus on the demarcation between exploration and confirmation, we encourage researchers to assess original pre-registration documents when evaluating publications, which is not a common practice [15]. Early consideration of whether research is exploratory or confirmatory should allow researchers to choose which publication format enables them to report their research process accurately and honestly.
